# Interaction of night shift work with polymorphism in melatonin receptor 1B gene on incident stroke

**DOI:** 10.5271/sjweh.4025

**Published:** 2022-06-30

**Authors:** Yilin Chen, Lulu Yang, Yannis Yan Liang, Zhixuan He, Qi-Yong H Ai, Wenqian Chen, Huachen Xue, Mingqing Zhou, Yu Wang, Huan Ma, Qingshan Geng

**Affiliations:** 1 School of Medicine, South China University of Technology, Guangzhou, Guangdong, China; 2 Guangdong Cardiovascular Institute, Guangdong Provincial People’s Hospital, Guangdong Academy of Medical Sciences, Guangzhou, Guangdong, China; 3 Guangdong Mental Health Center, Guangdong Provincial People’s Hospital, Guangdong Academy of Medical Sciences, Guangzhou, Guangdong, China; 4 Department of Neurology, the First Affiliated Hospital, Jinan University, Guangzhou, Guangdong, China; 5 School of Medicine, Jinan University, Guangzhou, Guangdong, China; 6 Department of Health Technology and Informatics, The Hong Kong Polytechnic University, Hung Hom, Kowloon, Hong Kong SAR; 7 Department of Neurology, Yulin No.1 People’s Hospital, Yulin, Guangxi, China; 8 The Second School of Clinical Medicine, Southern Medical University, Guangzhou, Guangdong, China.; 9 Guangdong Provincial Key Laboratory of Coronary Heart Disease Prevention, Guangdong Cardiovascular Institute, Guangdong Provincial People’s Hospital, Guangdong Academy of Medical Sciences, Guangzhou, Guangdong, China

**Keywords:** circadian rhythm, MTNR1B rs10830963, stroke, UK Biobank

## Abstract

**Objectives:**

The aim of this study was to investigate whether melatonin receptor type 1B (MTNR1B) rs10830963 polymorphism interacts with night shift work on the risk of incident stroke.

**Methods:**

This study included individuals free of stroke at baseline from the UK Biobank. Night-shift work was assessed by the self-reported questions. MTNR1B rs10830963 was directly genotyped (CC, GC, and GG). Incident stroke was ascertained through hospital records and death registries. Cox proportional hazards models were employed to examine the associations of night shift work and MTNR1B rs10830963 with the risk of incident stroke.

**Results:**

A total of 242 194 participants were finally included (mean age: 52.95 years; 51.63% women). Over 12-year follow-up, 3287 incident stroke events occurred. Night shift work increased the risk of incident stroke [hazard ratio (HR) 1.13, 95% confidence interval (CI) 1.00–1.28] after adjusting for socio-demographics, and this association attenuated after additional adjustment for lifestyle factors (HR 1.06, 95% CI 0.94–1.20). MTNR1B rs10830963 polymorphism modified the association between night shift work and incident stroke (P_for interaction_ =0.010). In the Cox models adjusted for socio-demographics and lifestyle factors, among night-shift workers, minor allele G was associated with a reduced risk of incident stroke (GC versus CC, HR 0.74, 95% CI 0.58–0.95; GG versus CC, HR 0.65, 95% CI 0.40–1.06; P_for trend_=0.010); while night shift work was associated with a higher stroke risk only among MTNR1B rs10830963 CC carriers (HR 1.23, 95% CI 1.05–1.44) but not GC/GG carriers.

**Conclusions:**

These results suggest that MTNR1B rs10830963 may potentially modify the associations between night shift work and incident stroke.

Stroke is a worldwide leading cause of mortality and disability (1). In 2009, the Nurses’ Health Study (NHS)reported that rotating night shift work is an emerging modifiable risk factor of ischemic stroke, with a 4% increased risk for every five years of shift work among women nurses (2). In contrast, a recent meta-analysis finds an absence of association between shift work and stroke (3). Individual heterogeneity intolerance to night shift work determined by various factors, including gene-environment interaction, may partly explain the inconsistent findings (4).

Emerging evidence suggests that misalignment between internal (genetically determined clock) and external (dark-light cycle) circadian rhythms may serve as the biological mechanism linking increased risk of stroke and night shift work (5, 6). Melatonin is the essential hormone regulating the internal circadian rhythm released by the pineal gland. Recently, melatonin has been found to protect against vascular diseases like stroke and myocardial infarction mainly due to its antioxidative and anti-apoptosis effects (7, 8). Biologically, melatonin secretion follows a diurnal pattern with elevated secretion during nighttime while suppressed secretion during the day (9); therefore, it is plausible that the beneficial effects of melatonin may attenuate during night shift work when the day-night cycle is reversed.

On the other hand, the melatonin effects mainly depend on activating one of the two high-affinity membrane receptors, the melatonin receptor 1B (MTNR1B) (10). The polymorphism rs10830963 encodes MTNR1B (11). Notably, the MTNR1B rs10830963 minor allele G, compared with the standard C allele, is associated with substantially elevated expression of MTNR1B mRNA, leading to a higher level of melatonin signaling (11). Further, minor allele G is observed to be correlated with a dramatically more prolonged duration of elevated melatonin levels and delayed circadian phase of dim-light melatonin offset, namely, a delayed biologically dark-light cycle (12). As such, it is speculated that the G carriers possibly exhibit better synchrony between the delayed melatonin phase and the reverse external dark-light cycle attributed to night shift work.

Taken together, it is reasonable to argue that MTNR1B rs10830963 polymorphism may interact with night shift work to affect the incidence of stroke. Thus, the present study aimed to explore whether MTNR1B rs10830963 polymorphism interacted with night shift work to modify the risk of incident stroke in a population-based cohort study using the UK Biobank.

## Methods

Data from the UK Biobank study used in this study is available upon request from the UK Biobank data access committee (www.ukbiobank.ac.uk/enable-your-research/apply-for-access).

### Study setting and population

**Figure 1:**
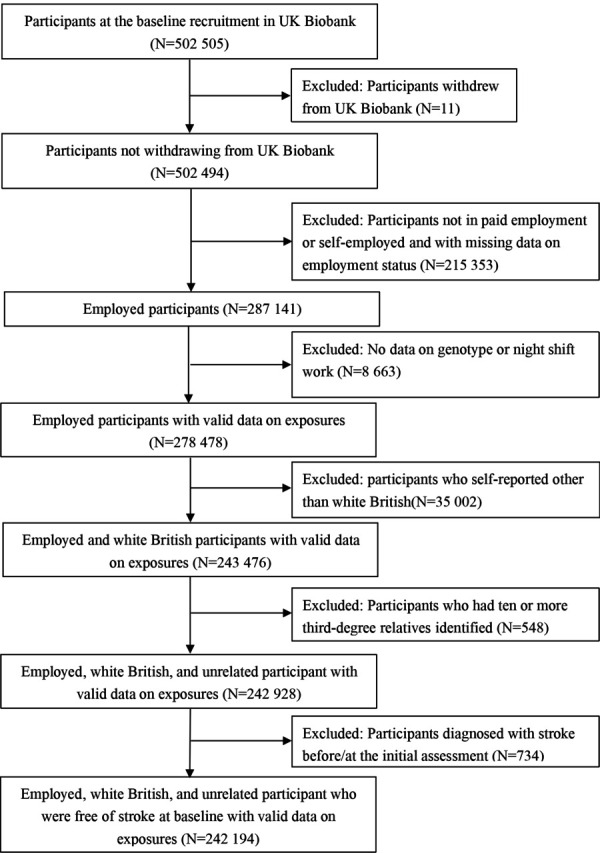
Flowchart of participant selection.

The UK Biobank is an ongoing, prospective, and population-based cohort. It included over 500 000 participants aged 40–69 years old from 22 assessment centers across the United Kingdom between 2006 and 2010. Participants completed detailed baseline assessments, including characterization of socio-demographics, lifestyles, medical history, and physical and functional measures if they consented to participate. Details of the rationale, design, and survey methods of UK Biobank have been described comprehensively online (www.UK biobank.ac.uk). The current study included the participants from UK Biobank who: (i) were employed with payment or self-employed, (ii) had available genotyping data, (iii) were of white British ancestry, (iv) did not have ≥10 third-degree relatives identified, and (v) were free of stroke at baseline. The current study’s flowchart of inclusion and exclusion is shown in figure 1.

### Exposure

*Night shift work*. In the UK Biobank, the employed status of each participant was queried once at baseline. Those with paid employment and shift work were further asked, “Does your work involve night shifts?” (Field ID 3426) with responses of (i) never/rarely, (ii) sometimes, (iii) usually, (iv) always, (v) do not know, or (vi) prefer not to answer. Night shift referred to working through the normal sleeping hours from 24:00–06:00 hours. The participants with responses of either “sometimes”, “usually”, or “always” were treated as “night shift workers”, while those who chose “prefer not to answer” or “do not know” were treated as missing cases and thus excluded from the current analysis.

### Genotyping

Detailed methods for Genotyping were described in a prior study (13). Participants were genotyped on the Affymetrix UK Biobank Lung Exome Evaluation Axiom array (Thermo Fisher Scientific, Santa Clara, California) or the Applied Biosystems UK Biobank Axiom Array (Thermo Scientific). MTNR1B rs10830963 (chromosome 11, intron variant) is amongst the directly genotyped single-nucleotide polymorphisms (SNP) of the UK Biobank. Quality control and imputation were conducted centrally using the Haplotype Reference Consortium, UK10K, and 1000 Genomes phase 3 reference panels. Testing for Hardy-Weinberg equilibrium revealed that the SNP did not deviate from the expected genotype proportion.

### Outcome

The primary outcome was any stroke including both ischemic and hemorrhagic strokes. Incident stroke was ascertained via linkage codes of hospital admissions and death registries in the UK Biobank. Detailed information regarding the linkage procedure is available online (biobank.ctsu.ox.ac.uk/showcase/label.cgi?id=2000). We used the International Classification of Diseases edition 10 codes to define stroke (I60–I64, I69). Participants diagnosed with stroke before/at baseline assessment were excluded from the current study. The follow-up for the study began at recruitment and ended at stroke diagnosis (for the participants with stroke) or at the time of death or censorship on 2 February 2021 (the latest date of complete coverage across hospital admissions and death registries used for UK Biobank data at the time of analysis), whichever came first.

### Covariates

We considered the following factors as the potential confounders, including age (Field ID 21022), sex (Field ID 31), education (highest qualification they owned) (Field ID 6138), location derived from assessment center (Field ID 54), Townsend Deprivation Index reflecting area-level socioeconomic deprivation (Field ID 189), smoking status (Field ID 20116), and alcohol intake frequency (Field ID 1558). Total sedentary time was estimated from the sum of self-reported hours spent watching television (Field ID 1170–1070), using the computer (Field ID 1180–1080), and driving (Field ID 1190–1090) during a typical day. Healthy diet score was calculated by using the following factors: vegetable intake (Field ID 1289 and 1299) ≥4 tablespoons each day (median); fruits intake (Field ID 1309 and 1319) ≥2.5 pieces each day (median); fish intake (Field ID 1329 and 1339) twice a week (median); unprocessed red meat intake (Field ID 1369, 1379, and 1389) twice a week (median); and processed meat intake (Field ID 1349) no more than twice each week (median). Each one point was given for each favorable diet factor, with the total diet score ranging from 0–5. In the sensitivity analysis, we further considered obesity, sleep duration, and insomnia as potential confounding factors. Obesity was defined by a body mass index (Field ID 21001) ≥30 kg/m^2^. Data on sleep duration (Field ID 1160) were collected by asking, “About how many hours sleep do you get in every 24 hours? (Please include naps)”. Given previously established nonlinear relationships with health (14), we categorized sleep duration into three groups: short (<6 hours), normal (6–8 hours), and long (>8 hours) sleep duration. Data on insomnia (Field ID 1200) were collected by asking, “Do you have trouble falling asleep at night or do you wake up in the middle of the night?” with responses of (i) never/rarely, (ii) sometimes, (iii) usually, (iv) prefer not to answer.

### Statistical analysis

Data were shown as mean ± standard deviation (SD), or proportion as appropriate. In total, 3685/242 194 (1.52%) participants had missing data for at least one covariate. Cox proportional hazards models were used to estimate the hazard ratios (HR) and 95% confidence intervals (CI) for incident stroke outcome, with night shift work, MTNR1B rs10830963 polymorphism, and their interaction as the exposures, respectively. The proportional hazards assumption for the Cox model was tested using the Schoenfeld residuals method and was satisfied. Two Cox proportional hazards models were performed with days of follow-up as the time-varying covariate. Participants with missing data were excluded from each model. Model 1 was adjusted for age (continuous), sex (women/men), socio-demographics including education (college or university degree/other), location (England/Wales/Scotland), and Townsend Deprivation Index (continuous). Model 2 was additionally adjusted for smoking status (never/previous/current), alcohol intake frequency (never or special occasions only, 1–3 or ≥3 times/week), total sedentary time (continuous), and healthy diet score (continuous). We constructed a cumulative incidence curve for the event of interest using the six quartiles categorized by MTNR1B rs10830963 polymorphism and night shift work status in the multivariable Cox model.

To examine the robustness of our findings, we performed some sensitivity analyses. First, we additionally controlled for obesity (yes/no), sleep duration (<6, 6–8, or >8 hours/day), and insomnia symptoms (never or rarely/sometimes/usually) based on model 2. Second, we repeated the main analyses by additionally including the sample of non-white British ancestry. The level of significance was set at P<0.05 (two-tailed). All statistical analyses were performed using the SPSS, version 26 (IBM Corp, Armonk, NY, USA).

## Results

From 502 494 participants enrolled between 2006 and 2010, we excluded 260 300 individuals who did not meet the inclusion criteria, leaving 242 194 participants in the final sample. Table 1 displays the baseline characteristics of the employed participants, grouped by night shift work status. Of the study sample, the mean age was 52.95 (SD 7.07) years, and 51.63% were women. A total of 19 692 (8.13%) employed participants reported being involved in night shift works. The proportions of “sometimes night shift workers”, “usually night shift workers”, and “always night shift workers” were 56.45%, 15.20%, and 28.35%, respectively. The distribution of MTNR1B rs10830963 was 52.47% for CC, 39.91% for GC, and 7.63% for GG. Briefly, compared with non-night shift workers, night shift workers had a trend to be younger, male, current smokers, drink less, sit more, and have a lower level of socioeconomic status and education (table 1). In addition, there was a greater proportion of Wales and Scotland population and higher stroke incidence among night shift workers (table 1). Further comparisons of the baseline characteristics according to the night shift work status and MTNR1B rs10830963 polymorphism are shown in the supplementary material https://www.sjweh.fi/article/4025, table S1.

**Table 1:** Demographic and clinical characteristics of the employed participants in the UK Biobank. [SD=standard deviation.]

Characteristics	Total sample (N=242 194)		Non-night shift (N=222 502)		Night shift (N=19 692)	
		
	N (%)	Mean (SD)	N (%)	Mean (SD)	N (%)	Mean (SD)
Age (years)		52.95 (7.07)		53.08 (7.07)		51.46 (6.82)
Sex (women)	125 056 (51.63)		117 872 (52.98)		7184 (36.48)	
Location						
England	213 065 (87.97)		195 956 (88.07)		17 109 (86.88)	
Wales	10 820 (4.47)		9820 (4.41)		1000 (5.08)	
Scotland	18 309 (7.56)		16 726 (7.52)		1583 (8.04)	
Townsend Deprivation Index ^a^		-1.56 (2.87)		-1.62 (2.84)		-0.91 (3.11)
Education (college or above)	87 576 (36.16)		84 209 (38.06)		3367 (17.25)	
Smoking status						
Never	138 282 (57.10)		128 310 (57.81)		9972 (50.82)	
Previous	78 068 (32.23)		71 738 (32.32)		6330 (32.26)	
Current	25 237 (10.42)		21 915 (9.87)		3322 (16.93)	
Alcohol intake						
Never or occasionally	33 578 (13.86)		30 348 (13.65)		3230 (16.42)	
1–2 times per week or 1–3 times per month	97 085 (40.09)		88 287 (39.70)		8798 (44.71)	
>3 times per week	111 419 (46.00)		103 771 (46.66)		7648 (38.87)	
Total sedentary time (hours)		4.74 (2.49)		4.67 (2.42)		5.50 (3.02)
Healthy diet score		2.70 (1.19)		2.72 (1.19)		2.53 (1.22)
Outcome						
Incident stroke	3287 (1.36)		2986 (1.34)		301 (1.53)	

a Calculated based on the preceding national census output areas before participants joined UK Biobank. Each participant is assigned a score corresponding to their postcode location, with a lower score indicating a lower level of social deprivation.

During a median follow-up of 12.0 years, 3287 (1.36%) participants developed a stroke. Night shift work significantly predicted incident stroke (HR 1.13, 95% CI 1.00–1.28) after adjusting for socio-demographics, including age, sex, education, location, and Townsend Deprivation Index; this association attenuated (HR 1.06, 95% CI 0.94–1.20) after additionally adjusting for smoking status, alcohol intake frequency, total sedentary time, and healthy diet score (supplementary table S2). However, MTNR1B rs10830963 polymorphism alone was not significantly associated with incident stroke (GC versus CC: HR 0.99, 95% CI 0.92–1.06; GG versus CC: HR 0.96, 95% CI 0.84–1.10) (data not shown).

**Figure 2:**
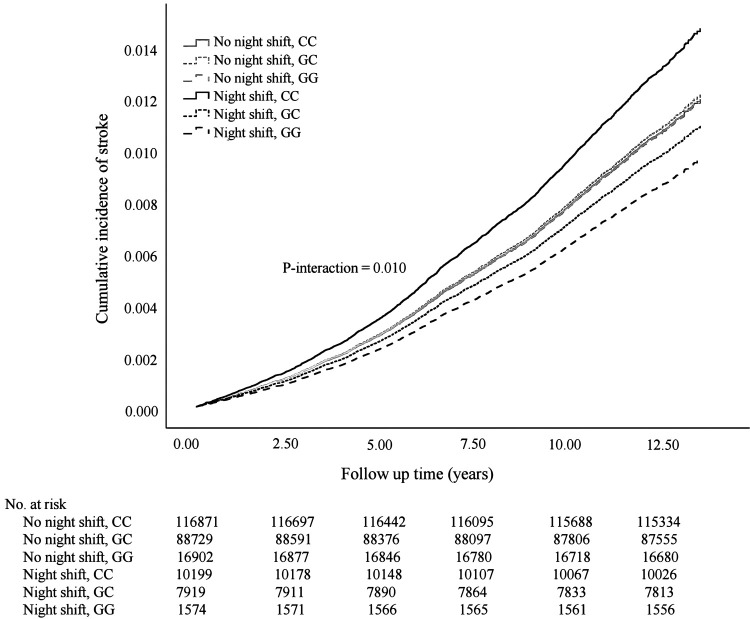
Cumulative incidence curve for incident stroke according to night shift work status and MTNR1B rs10830963 polymorphism in multivariable Cox model. Multivariable Cox model was adjusted for age, sex, education, location, Townsend Deprivation Index , smoking status, alcohol intake frequency, total sedentary time, and healthy diet score. The P-value indicated interaction between night shift work status and MTNR1B rs10830963 polymorphism in the multivariable Cox model.

A significant interaction was found between MTNR1B rs10830963 polymorphism and night shift work on the risk of developing stroke in the multivariable Cox model (P_for interaction_=0.010, figure 2). Among night shift workers rather than non-night shift workers, minor allele G became a protective polymorphism against incident stroke (GC versus CC, HR 0.76, 95% CI 0.60–0.97; GG versus CC, HR 0.66, 95% CI 0.40–1.07; P_for trend_=0.012, model 1), and these associations remained largely unchanged after further controlling for lifestyle factors (table 2, model 2). When stratified by MTNR1B rs10830963 genotypes, night shift work increased the risk of incident stroke only among those with MTNR1B rs10830963 CC (HR 1.23, 95% CI 1.05–1.44) but not GC/GG genotype (table 3, model 2). In the sensitivity analyses, further adjustment for obesity, sleep duration, and insomnia did not substantially change the association (GC versus CC, HR 0.74, 95% CI 0.58–0.94; GG versus CC, HR 0.67, 95% CI 0.41–1.09). A significant positive trend still existed between minor allele G and stroke risk among the night shift workers (P_for trend_=0.012) (supplementary table S3). Particularly, night shift work was still associated with a higher risk only among those with MTNR1B rs10830963 CC but not GC/GG genotype (supplementary table S4). Moreover, the main results did not substantially change after additionally including the sample of non-white British ancestry (supplementary table S5).

**Table 2:** Cox regression analyses of MTNR1B rs10830963 variants and night shift work on incident stroke among employed participants in the UK Biobank. [CI=confidence interval; HR=hazard ratios; MTNR1B=melatonin receptor type 1B.]

Night shift work status	rs10830963 genotypes	Incident stroke	

		Model 1 ^a^ HR (95% CI)	Model 2 ^b^ HR (95% CI)
Non-nightshift	Stroke cases (N)/person-years	2 949/2 650 270	2 939/2 642 252
	CC	1.00	1.00
	GC	1.01 (0.94–1.09)	1.01 (0.94–1.09)
	GG	1.00 (0.87–1.15)	1.00 (0.86–1.15)
	P for trend	0.84	0.86
Night shift	Stroke cases (N)/person-years	300/233 700	298/232 454
	CC	1.00	1.00
	GC	0.76 (0.60–0.97)	0.74 (0.58–0.95)
	GG	0.66 (0.40–1.07)	0.65 (0.40–1.06)
	P for trend	0.012	0.010

a Model 1 adjusted for age, sex, education, location, and Townsend Deprivation Index.

b Model 2 adjusted for age, sex, education, location, Townsend Deprivation Index, smoking status, alcohol intake frequency, total sedentary time, and healthy diet score.

**Table 3:** Association of night shift work with incident stroke stratified by MTNR1B rs10830963 polymorphism among employed participants in the UK Biobank. [CI=confidence interval; HR=hazard ratios; MTNR1B=melatonin receptor type 1B.]

rs10830963 genotypes	Night shift work status	Incident stroke	

		Model 1 ^a^ HR (95% CI)	Model 2 ^b^ HR (95% CI)
CC	Stroke cases (N)/person-years	1721/1 512 775	1715/1 507 743
	Non-night shift	1.00	1.00
	Night shift	1.30 (1.11-1.52)	1.23 (1.05-1.44)
GC	Stroke cases (N)/person-years	1288/1 151 266	1284/1 147 746
	Non-night shift	1.00	1.00
	Night shift	0.97 (0.80-1.19)	0.90 (0.74-1.10)
GG	Stroke cases (N)/person-years	240/219 929	238/219 217
	Non-night shift	1.00	1.00
	Night shift	0.85 (0.53-1.38)	0.81 (0.50-1.31)

a Model 1 adjusted for age, sex, education, location, and Townsend Deprivation Index.

b Model 2 adjusted for age, sex, education, location, Townsend Deprivation Index, smoking status, alcohol intake frequency, total sedentary time, and healthy diet score.

## Discussion

This population-based cohort study confirmed that night shift work was potentially a modifiable risk factor of incident stroke over the 12-year follow-up. More importantly, we found that MTNR1B rs10830963 modified the risks of night shift work on incident stroke. Particularly, night shift work was most risky to predispose to stroke occurrence among the CC genotype carriers in MTNR1B rs10830963; while among the night shift workers, the G allele seemed to be a protective allele against the detrimental effects of night shift work on incident stroke.

The finding that night shift work potentially increased stroke risk was consistent with the prior analyses of the NHS (2, 15). In the population-based cohort of NHS, Brown, et al (2) found that the duration of rotating night shift work among the nurses showed a linear trend with risk of ischemic stroke. Recently, a study re-assessed the association between rotating night shifts and the risk of coronary heart disease using data from NHS and NHS2 with over 20 years follow-up and reported that longer duration of rotating night shift work modestly increased the risk of coronary heart disease among women nurses (15). Although a recent meta-analysis reported a null association, most of its five studies included were subject to limitations, including small sample size and cross-sectional design (3). It is suggested that rotating night shift work may inhibit melatonin secretion (6). Since melatonin regulates the circadian rhythm, the disinhibition of melatonin secretion may result disrupt circadian rhythm, which has widely been demonstrated to be associated with multiple vascular and metabolic disorders (2, 6, 15, 16).

To date, there have been rare studies about the association of MTNR1B rs10830963 polymorphism with incident stroke. To the best of our knowledge, this study was the first to reveal that the MTNR1B rs10830963 G allele alleviated adverse effects of night shift work on stroke. Despite the lack of direct evidence, similar to our finding, a recent study demonstrated a pattern that MTNR1B rs10830963 interacted with night shift work to modify the risk of incident prostate cancer (17). An earlier study also demonstrated that the MTNR1B rs10830963 G allele represented in haplotype 2 was associated with better cardiac function in patients with hypertension (18). However, another study observed no interactive effects of MTNR1B rs10830963 and shift work on diabetes risk (19). In addition, our study found that the interaction of night shift work, MTNR1B rs10830963, with incident stroke, slightly attenuated after adjusting for lifestyle factors. This finding suggests that lifestyle factors like smoking, alcohol consumption, and diet may serve as potential mediators of such associations.

Several lines of mechanisms may explain the modification effect of MTNR1B rs10830963 on stroke occurrence. First, the MTNR1B rs10830963 G allele was related to a delayed melatonin secretion phase and a later sleep-timing and caloric-intake pattern, suggesting an evening circadian preference (12, 20). Our results, therefore, implicated that better synchrony of the internal (melatonin-mastered clock) and external (reverse dark-light cycle by night shift work) clock cycles reduced the risk of incident stroke. Consistently, an experimental study showed that misalignment of the animal’s internal clock cycle and external cycle using genetic mutations had led to cardiovascular dysfunction (6). On the other hand, melatonin exerts pleiotropic neuroprotective effects by reducing oxidative/inflammatory stress or enhancing neurogenesis (8). MTNR1B rs10830963 G allele was correlated with higher melatonin signaling by increasing the expression of MTNR1B, which subsequently enhances the protective effects of melatonin. An alternative explanation centers on the finding that stroke occurrence often peaks in the morning (6, 12). In parallel, MTNR1B rs10830963 G allele carriers commonly show elevated melatonin secretion in the morning. Due to the vascular protective effects of melatonin, MTNR1B rs10830963 G carriers are therefore suggested to have a higher tolerance to the vascular lesions peaking in the morning (6, 8, 12, 19).

### Strengths and limitations

The present study had strengths, including a large sample size, long follow-up duration, and consideration of the genetic susceptibility to night shift work. However, several limitations are noteworthy. First, this study was subject to residual confounding factors, for example, the data of some factors influencing circadian rhythm like light exposure, dietary behaviors, and physical activity during night shift work was lacking, which may have confounded our results. We extensively adjusted for multiple covariates involved in lifestyle factors affecting stroke in the Cox regression models. Nonetheless, there may still be some other residual factors that we have not considered in this study. Second, the present study lacked the information about the duration and intensity of night shift work at the baseline to further clarify the dose–response association between night shift work and incident stroke risk. Third, we were unable to exclude individuals who occasionally took night shift work, due to a lack of specific definitions about the responses of questions on shift work (for instance, “sometimes” and “usually”) in the UK Biobank. As such, the associations may have been overestimated. Fourth, this longitudinal study excluded the individuals with missing data, which could have led to selection bias, although the magnitude of missing data was not large. Fifth, stroke cases were ascertained only by hospital admissions records and death registries in the present study. The milder cases without hospital admissions may be underestimated. In this case, the associations of night shift work, MTNR1B rs10830963 polymorphism, and incident stroke may be undervalued, namely, the results may be towards null. Last, the study sample of the UK Biobank were mainly middle-aged adults, of which about half (57.1%) were currently employed. As such, the association between night shift work and incident stroke could be underrated due to healthy-worker effects. The present study’s findings may not be generalized to the younger population.

### Concluding remarks

In summary, the risk of night shift work on incident stroke was potentially modified by MTNR1B rs10830963. In addition, due to the modifiable effects of MTNR1B rs10830963, melatonin supplementation may serve as a potential strategy to reduce stroke risk, particularly for the MTNR1B rs10830963 CC genotype carriers when night shifts are inevitably involved.

## Supplementary material

Supplementary material

## Data Availability

All data included in this study are publicly available via the platform of UK Biobank (https://www.ukbiobank.ac.uk/).

## Protection of research participants

The UK Biobank received ethical approval from the NHS (National Health Service) National Research Ethics Service (16/NW/0274). All UK Biobank study participants provided informed consent.
