# Supplementary material for: Interaction of night shift work with polymorphism in melatonin receptor 1B gene on incident stroke
Source: Scand J Work Environ Health. 2022 Jun 30;48(5):372–9. doi: 10.5271/sjweh.4025 (PMC9527780; doi:10.5271/sjweh.4025)
Supplement: Supplementary material [file SJWEH-48-372-S001.pdf]

Interaction of night shift work with polymorphism in melatonin receptor 1B gene on incident stroke[1]

by Yilin Chen, MD, Lulu Yang, MD, PhD, Yan Liang, MD, PhD, Zhixuan He, Qi-Yong H Ai, MD, PhD, Wenqian Chen, MD, Huachen Xue, MSc, Mingqing Zhou, MD, Yu Wang, MD, Huan Ma, MD, PhD, Qingshan Geng, MD, PhD [2]

1. *Supplementary material*
2. *Correspondence to: Qingshan Geng, MD, PhD, Guangdong Cardiovascular Institute, Guangdong Provincial People's Hospital, Guangdong Academy of Medical Sciences, Guangzhou, Guangdong, China, 102 Zhongshan Road, Guangzhou, Guangdong, China; or School of Medicine, South China University of Technology, Guangzhou, Guangdong, China. [E-mail: gengqingshan@gdph.org.cn]*

**Supplementary Table S1**	Demographic and clinical characteristics of the employed participants stratified by night shift work and MTNR1B rs10830963 polymorphism in the UK Biobank. [BMI, body mass index; SD=standard deviation.]
**Supplementary Table S2**	Association of night shift work with incident stroke among employed participants in the UK Biobank. [CI=confidence interval; HR=hazard ratios; MTNR1B=melatonin receptor type 1B.]
**Supplementary Table S3**	Sensitivity analysis on the association of MTNR1B rs10830963 variants with incident stroke stratified by night shift work status among employed participants in the UK Biobank after further adjustment for obesity and sleep traits. [CI=confidence interval; HR=hazard ratios; MTNR1B=melatonin receptor type 1B.]
**Supplementary Table S4**	Sensitivity analysis on the association of night shift work with incident stroke stratified by MTNR1B rs10830963 polymorphism among employed participants in the UK Biobank after further adjustment for obesity and sleep traits. [CI=confidence interval; HR=hazard ratios; MTNR1B=melatonin receptor type 1B.]
**Supplementary Table S5**	Sensitivity analysis on the association of MTNR1B rs10830963 variants and night shift work with incident stroke among employed participants in the UK Biobank after including the sample of non-white British ancestry. [CI=confidence interval; HR=hazard ratios; MTNR1B=melatonin receptor type 1B.]

Supplementary Table S1. Demographic and clinical characteristics of the employed participants stratified by night shift work and MTNR1B rs10830963 polymorphism in the UK Biobank. [BMI, body mass index; SD=standard deviation.]

Characteristics	GG				GC				CC			
	Non-night Shift (N=16 902)		Night Shift (N=1 574)		Non-night Shift (N=88 729)		Night Shift (N=7 919)		Non-night Shift (N=116 871)		Night Shift (N=10 199)	
	N (%)	Mean (SD)	N (%)	Mean (SD)	N (%)	Mean (SD)	N (%)	Mean (SD)	N (%)	Mean (SD)	N (%)	Mean (SD)
Age (years)		53.03 (7.02)		51.42 (6.80)		53.07 (7.08)		51.58 (6.82)		53.11 (7.07)		51.37 (6.82)
Sex (Women)	8 897 (52.64)		555 (35.26)		47 107 (53.09)		2 888 (36.47)		61 868 (52.94)		3 741 (36.68)	
Location												
England	14 862 (87.93)		1 362 (86.53)		77 931 (87.83)		6 849 (86.49)		103 163 (88.27)		8 898 (87.24)	
Wales	800 (4.73)		90 (5.72)		4 058 (4.57)		422 (5.33)		4 962 (4.25)		488 (4.78)	
Scotland	1 240 (7.34)		122 (7.75)		6 740 (7.60)		648 (8.18)		8 746 (7.48)		813 (7.97)	
Townsend Deprivation Index [a]		-1.61 (2.85)		-1.02 (3.05)		-1.63 (2.84)		-0.86 (3.11)		-1.62 (2.84)		-0.93 (3.11)
Education (College or above)	6 382 (37.76)		248 (15.76)		33 542 (37.80)		1 372 (17.33)		44 285 (37.89)		1 747 (17.13)	
Smoking status												
Never	9 621 (56.92)		781 (49.62)		51 299 (57.82)		4 053 (51.18)		67 390 (57.66)		5138 (50.38)	
Previous	5 507 (32.58)		525 (33.35)		28 536 (32.16)		2 493 (31.48)		37 695 (32.25)		3 312 (32.47)	
Current	1 731 (10.24)		264 (16.77)		8 687 (9.79)		1 347 (17.01)		11 497 (9.84)		1 711 (16.78)	
Alcohol intake												
Never or occasionally	2 311		240		12 135		1 290		15 902		1 700	

	(13.67)	(15.25)	(13.68)	(16.29)	(13.61)	(16.67)
1-2 times per week or 1-3 times per month	6 705 (39.67)	711 (45.17)	35 286 (39.77)	3 527 (44.54)	46 296 (39.61)	4 560 (44.71)
>3 times per week	7 878 (46.61)	623 (39.58)	41 280 (46.52)	3 095 (39.08)	54 613 (46.73)	3 930 (38.53)
Total sedentary time (hours)	4.66 (2.41)	5.51 (2.93)	4.68 (2.44)	5.53 (3.06)	4.66 (2.41)	5.47 (3.00)
Healthy diet score	2.72 (1.18)	2.58 (1.24)	2.72 (1.19)	2.53 (1.23)	2.72 (1.19)	2.53 (1.21)
Obesity (BMI ≥30)	3 897 (23.06)	500 (31.77)	20 138 (22.70)	2 439 (30.80)	25 965 (22.22)	3 078 (30.18)
Sleep duration						
<6 h per day	728 (4.31)	121 (7.69)	3 766 (4.24)	675 (8.52)	4 936 (4.22)	814 (7.98)
6-8 h per day	15 405 (91.14)	1 352 (85.90)	80 892 (91.17)	6 802 (85.89)	106 592 (91.20)	8 793 (86.21)
>8 h per day	730 (4.32)	83 (5.27)	3 874 (4.37)	387 (4.89)	5 025 (4.30)	525 (5.15)
Insomnia						
Never/rarely	4 630 (27.39)	438 (27.83)	24 125 (27.19)	2 185 (27.59)	31 685 (27.11)	2 794 (27.39)
Sometimes	8 039 (47.56)	751 (47.71)	42 484 (47.88)	3 683 (46.51)	55 904 (47.83)	4 865 (47.70)
Usually	4 226 (25.00)	381 (24.21)	22 095 (24.90)	2 035 (25.70)	29 245 (25.02)	2 511 (24.62)
Outcome						
Incident stroke	228 (1.35)	18 (1.14)	1 193 (1.34)	107 (1.35)	1 565 (1.34)	176 (1.73)

[a] Calculated based on the preceding national census output areas before participants joined UK Biobank. Each participant is assigned a score corresponding to their postcode location, with a lower score indicating a lower level of social deprivation.

Supplementary Table S2. Association of night shift work with incident stroke among employed participants in the UK Biobank. [CI=confidence interval; HR=hazard ratios; MTNR1B=melatonin receptor type 1B.]

Night shift work status	Incident stroke	
	**Model 1 [a]<br>HR (95% CI)**	**Model 2 [b]<br>HR (95% CI)**
Stroke cases (N)/person-years	3 249/2 883 970	3 237/2 874 706
Non-night Shift	1.00	1.00
Night Shift	1.13 (1.00-1.28)	1.06 (0.94-1.20)

[a] Model 1 adjusted for age, sex, education, location, and Townsend Deprivation Index.

[b] Model 2 adjusted for age, sex, education, location, Townsend Deprivation Index, smoking status, alcohol intake frequency, total sedentary time, and healthy diet score.

Supplementary Table S3. Sensitivity analysis on the association of MTNR1B rs10830963 variants with incident stroke stratified by night shift work status among employed participants in the UK Biobank after further adjustment for obesity and sleep traits. [CI=confidence interval; HR=hazard ratios; MTNR1B=melatonin receptor type 1B.]

**Night shift work status**	**rs10830963 genotypes**	**Incident stroke**	
		**Age and sex-adjusted HR (95% CI)**	**Full adjusted [a] HR (95% CI)**
Non-night Shift	Stroke cases (N)/person-years	2 986/2 669 660	2 917/2 630 791
	CC	1.00	1.00
	GC	1.01 (0.94-1.09)	1.01 (0.94-1.09)
	GG	1.02 (0.88-1.17)	1.00 (0.87-1.15)
	P for trend	*0.79*	*0.85*
Night Shift	Stroke cases (N)/person-years	301/236 142	293/230 197
	CC	1.00	1.00
	GC	0.77 (0.60-0.97)	0.74 (0.58-0.94)
	GG	0.66 (0.40-1.06)	0.67 (0.41-1.09)
	P for trend	*0.012*	*0.012*

[a] Full model adjusted for age, sex, education, location, Townsend Deprivation Index, smoking status, alcohol intake frequency, total sedentary time, healthy diet score, obesity, sleep duration, and insomnia.

Supplementary Table S4. Sensitivity analysis on the association of night shift work with incident stroke stratified by MTNR1B rs10830963 polymorphism among employed participants in the UK Biobank after further adjustment for obesity and sleep traits. [CI=confidence interval; HR=hazard ratios; MTNR1B=melatonin receptor type 1B.]

rs10830963 genotypes	Night shift work status	Incident stroke	
		**Age and sex-adjusted HR (95% CI)**	**Full adjusted [a] HR (95% CI)**
CC	Stroke cases (N)/person-years	1 741/1 524 599	1 700/1 500 255
	Non-night Shift	1.00	1.00
	Night Shift	1.39 (1.19-1.63)	1.21 (1.04-1.42)
GC	Stroke cases (N)/person-years	1 300/1 159 433	1 272/1 142 574
	Non-night Shift	1.00	1.00
	Night Shift	1.06 (0.87-1.29)	0.88 (0.72-1.08)
GG	Stroke cases (N)/person-years	246/221 771	238/218 159
	Non-night Shift	1.00	1.00
	Night Shift	0.90 (0.56-1.45)	0.81 (0.50-1.31)

[a] Full model adjusted for age, sex, education, location, Townsend Deprivation Index, smoking status, alcohol intake frequency, total sedentary time, healthy diet score, obesity, sleep duration, and insomnia.

Supplementary Table S5. Sensitivity analysis on the association of MTNR1B rs10830963 variants and night shift work with incident stroke among employed participants in the UK Biobank after including the sample of non-white British ancestry. [CI=confidence interval; HR=hazard ratios; MTNR1B=melatonin receptor type 1B.]

Night shift work status	rs10830963 genotypes	Incident stroke	
		**Model 1 [a] HR (95% CI)**	**Model 2 [b] HR (95% CI)**
Non-night Shift	Stroke cases (N)/person-years	3 323/2 998 028	3 309/2 988 096
	CC	1.00	1.00
	GC	1.02 (0.95-1.10)	1.02 (0.95-1.10)
	GG	1.02 (0.89-1.16)	1.01 (0.88-1.15)
	P for trend	*0.61*	*0.68*
Night Shift	Stroke cases (N)/person-years	367/288 219	365/286 314
	CC	1.00	1.00
	GC	0.83 (0.67-1.03)	0.81 (0.65-1.01)
	GG	0.66 (0.42-1.03)	0.66 (0.42-1.03)
	P for trend	*0.021*	*0.017*

[a] Model 1 adjusted for age, sex, education, location, and Townsend Deprivation Index.

[b] Model 2 adjusted for age, sex, education, location, Townsend Deprivation Index, smoking status, alcohol intake frequency, total sedentary time, and healthy diet score.
